# NMR Investigation of Water Molecular Dynamics in Sulfonated Polysulfone/Layered Double Hydroxide Composite Membranes for Proton Exchange Membrane Fuel Cells

**DOI:** 10.3390/membranes13070684

**Published:** 2023-07-22

**Authors:** Cataldo Simari

**Affiliations:** 1 Department of Chemistry and Chemical Technologies, University of Calabria, 87036 Rende, Italy; cataldo.simari@unical.it; Tel.: +39-0984-493385; Fax: +39-0984-492044; 2 National Reference Centre for Electrochemical Energy Storage (GISEL)—INSTM, Via G. Giusti 9, 50121 Firenze, Italy

**Keywords:** sulfonated polysulfone, LDH, nanocomposite membranes, water molecular dynamics, pulsed field gradient (PFG) NMR, proton conductivity

## Abstract

The development of nanocomposite membranes based on hydrocarbon polymers is emerging as one of the most promising strategies for overcoming the performance, cost, and safety limitations of Nafion, which is the current benchmark in proton exchange membranes for fuel cell applications. Among the various nanocomposite membranes, those based on sulfonated polysulfone (sPSU) and Layered Double Hydroxides (LDHs) hold promise regarding their successful utilization in practical applications due to their interesting electrochemical performance. This study aims to elucidate the effect of LDH introduction on the internal arrangement of water molecules in the hydrophilic clusters of sPSU and on its proton transport properties. Swelling tests, NMR characterization, and Electrochemical Impedance Spectroscopy (EIS) investigation allowed us to demonstrate that LDH platelets act as physical crosslinkers between -SO_3_H groups of adjacent polymer chains. This increases dimensional stability while simultaneously creating continuous paths for proton conduction. This feature, combined with its impressive water retention capability, allows sPSU to yield a proton conductivity of ca. 4 mS cm^−1^ at 90 °C and 20% RH.

## 1. Introduction

Proton exchange membrane fuel cells (PEMFCs) are on the verge of creating a vast revolutionary change in the field of electricity [1,2,3,4]. The outstanding potential of this technology relies on the ability to combine high energy conversion efficiency, rapid start-up times, high power density, energy supply, and, of no less significance, low emissions [5,6,7]. All the above factors have pushed PEMFCs to the top position among electrochemical-generating devices.

Unfortunately, three severe issues still limit their large-scale application: (i) the platinum anode catalyst employed in PEMFCs is easily poisoned by CO unless the operating temperature is increased above 100 °C; (ii) rapid water loss occurs with an elevated temperature; and (iii) the economic, safety, and environmental costs for the synthesis of the proton exchange membrane (PEM), which is one of the key components of these devices, are quite high [8,9,10]. Indeed, perfluorinate sulfuric acid polymers (e.g., Nafion, Aciplex, and Flemion), which still represent state-of-the art materials in relation to the current technology, are typically affected by a severe reduction in proton conductivity under high operating temperatures due to the dehydration of water from the membrane [11,12,13,14]. Furthermore, water evaporation also results in membrane shrinkage and the deterioration of the membrane/electrode interfaces, which, in turn, result in a dramatic reduction in the devices’ performance [10,15]. In summary, developing PEMs capable of efficiently operating in a wide range of different conditions, such as high-temperature and/or anhydrous conditions, represents, to date, one of the most compelling challenges in the field of PEMFCs.

Currently, the incorporation of inorganic nanoparticles inside the Nafion matrix has been the general strategy for improving water retention and proton conductivity [16]. In this regard, a multitude of fillers have been tested, including metal oxide particles [17,18,19], aluminosilicate compounds [20,21], alumina particles [22], graphene oxide [23,24], and heteropolyacids [25], which have demonstrated great improvements in terms of proton conductivity. Nevertheless, issues regarding cost, safety, and environmental impacts due to the perfluorinated backbone still persist. Consequently, there is a need to develop alternative PEMs that are cost-effective and ecofriendly.

In this regard, very interesting results have been achieved with the sulfonated derivatives of hydrocarbon polymers, such as sulfonated poly (arylene ether ketone) [26,27], sulfonated poly (ether ether ketone) [28,29,30], sulfonated polyimide [31,32], sulfonated polysulfone [33,34], and related composite membranes [35].

Among the plethora of potential combinations of hydrocarbon polymers and inorganic fillers, nanocomposite membranes based on sulfonated polysulfone (sPSU) and Layered Double Hydroxides (LDHs) have been attracting increasing interest due to their unique combination of thermal, mechanical, and transport features. In fact, sPSU offers the advantages of high commercial availability and low cost, superior chemical and thermomechanical stability, and an ecofriendly nature [33,36,37,38]. Furthermore, the properties of this polymer can be tuned by finely controlling the number of sulfonic acid groups grafted to the polymer backbone [39]. On the other side, Layered Double Hydroxides (LDH) nanoparticles belong to the anionic (or hydrotalcite-type) clay family with a chemical structure similar to that of brucite Mg(OH)_2_. The LDH structure has a layer-type lattice and can be represented as [M_1−x_^(II)^ M_x_^(III)^ (OH)_2_]^x+^[A^m−^_x/m_]·nH_2_O, where M^(II)^ is a divalent metal cation (such as Mg, Mn, Fe, Co, Ni, Cu, Zn, or Ga), M^(III)^ is a trivalent metal cation (such as Al, Cr, Mn, Fe, Co, Ni, or La), A^m−^ is an interlayer anion (such as CO_3_^2−^, OH^−^, NO_3_^−^, SO_4_^2−^, or ClO_4_^−^ [40,41,42]), and x is the molar ratio or layer charge density value (M^3+^/(M^2+^ + M^3+^). Therefore, the layers have a fixed positive charge, and neutrality is obtained via anions present in the interlamellar space. Such a peculiar structure provides unique physicochemical properties: easy synthesis and functionalization, high purity, the effective control of particle size and crystallinity, and very high ion exchange capacity (IEC) [43,44,45,46]. In 2014, Herrero and coworkers reported the first preparation of sPSU/LDH nanocomposite membranes, demonstrating that LDHs had a positive impact on the mechanical and conductivity properties of the resulting electrolyte [47,48]. Recently, we demonstrated that the incorporation of LDH material inside the sPSU matrix enables the preparation of very versatile nanocomposite PEMs that exceed benchmark performance in both high-temperature PEMFCs [49] and direct methanol fuel cells [50]. While a moderately sized body of literature exists regarding the electrochemical performance of sPSU/LDH membranes, there is a severe lack of information on the structure/performance relationship and the molecular phenomena occurring between the polymer matrix, LDH platelets, and water molecules for this electrolyte.

In this study, we propose an extensive NMR analysis of the water distribution and molecular dynamics in nanocomposite membranes based on sulfonated polysulfone and layered double hydroxides. A previous study demonstrated that optimal performance was achieved with a combination of sPSU with an of IEC 1.36 meq g^−1^ and a Mg^2+^/Al^3+^ LDH material (at a 2:1 metal ratio and with NO_3_^−^ as an interlayer anion, thus having empirical formula of [Mg_0.67_^(2+)^ Al_0.33_^(3+)^ (OH)_2_]^0.33+^[(NO_3_^−^)_0.33_]·3H_2_O) at 3% wt. of loading. Under these conditions, highly homogeneous and completely exfoliated membranes were obtained [49,50]. Consequently, the membranes in this study were prepared by maintaining the same composition and using the preparation procedure. ^1^H NMR spectroscopy was used to elucidate the internal arrangement of water molecules and molecular dynamics through spectral analysis, diffusometry, and relaxometry. The NMR data allowed us to clarify the effect of the LDH platelets on the water distribution in the sPSU polymer as a function of temperature. Such a systematic study, to the best of our knowledge, has not been reported in the published literature. Finally, electrochemical impedance spectroscopy (EIS) was carried out to measure the proton conductivity of the PEMs.

## 2. Materials and Methods

### 2.1. Materials and Chemicals

Commercial polysulfone (Lasulf, LATI Industria Termoplastici S.p.A, Vedano Olona (VA) Italy) was supplied by Lati SPA (Varese, Italy). Chloroform (anhydrous, ≥99%), trimethylsilyl chlorosulfonate (99%), sodium methoxide/methanol solution (30% wt.), ethanol (96%), and *N*, *N*-dimethylacetamide (DMAc, ReagentPlus^®^, 99%) were all purchased from Sigma Aldrich (Sigma-Aldrich, Milan, Italy) and used as received.

### 2.2. Synthesis of Sulfonated Polysulfone

Sulfonated polysulfone (sPSU) was synthesized according to the procedure reported by Lufrano et al. [51]. A total of 5 g of PSU was dried at 120 °C for 24 h. Thereafter, the polymer was dissolved in 100 mL of anhydrous chloroform at room temperature until a homogenous solution was obtained. The introduction of chloromethyl groups was achieved via treatment with trimethylsilyl chlorosulfonate for 6 h at 50 °C under reflux conditions followed by reaction with sodium methoxide/methanol solution (30% wt.) at 50 °C for 1 h. During this step, sulfonated polysulfone in sodium form was achieved, which was then recovered via precipitation in an ethanol bath, washed several times with deionized water, filtrated, and dried at 70 °C for 48 h. This procedure allowed us to synthesize sPSU with an ion exchange capacity of 1.36 meq g^−1^.

### 2.3. Synthesis of Layered Double Hydroxides

Mg^2+^/Al^3+^ LDH material with a metal ratio of 2:1 and employing NO_3_^−^ as an interlayer ion was synthesized according to a procedure reported in a previous paper [49]. Mg^2+^ and Al^3+^ nitrate salts were co-precipitated in an aqueous solution of NaOH (2.5 M) until a pH of 10 was reached. The procedure was carried out under inert atmosphere (N_2_ gas flow) to avoid any contamination. The resulting dispersion was then stirred for 6 h at 60 °C, and the LDH particles were then recovered via centrifugation. Finally, the powdered material was washed several times with distilled water and dried in an oven at 80 °C for one day.

### 2.4. Preparation of Sulfonated Polysulfone Membranes

**Figure 1:**
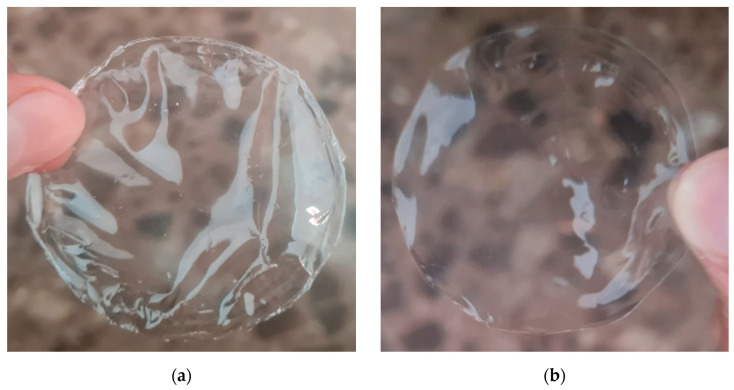
Photos of (**a**) sPSU and (**b**) sPL membranes.

Pristine membranes were prepared by dissolving an adequate amount of dry sPSU powder in *N*, *N*-dimethylacetamide (DMAc) at room temperature. After a homogeneous solution was obtained, this solution was poured onto a glass plate and heated at 55 °C until dry. For the preparation of composite membranes (sPL), the appropriate amount of LDH material was directly dispersed in a sPSU-DMAc polymer solution. The dispersion was left for at least 6 h under vigorous mechanical stirring alternating with sonication in an ultrasonic bath. Once the dispersion was macroscopically homogeneous, it was poured onto a glass plate and dried at 55 °C until complete evaporation of the solvents. For this study, membranes at 3% wt. of the filler loading with respect to the polymer mass were prepared. Both membranes were transparent, flexible, and macroscopically homogeneous (see Figure 1), with a dry thickness ranging between 50 and 55 μm. Before testing, both the sPSU and sPL membranes were treated with 1 M of H_2_SO_4_ solution at 55 °C for 15 h for chemical activation followed by being washed several times with distilled water to remove any residual acid.

### 2.5. Membranes’ Characterization

Conventional titration method was employed to determine the ion exchange capacity (IEC) of the PEMs [52]. The weight and volume variations between dry and wet states were used to calculate water uptake (*wu*) and volume swelling (Δ*V*), respectively. Specifically, rectangular samples were directly cut from dry membranes, their weight (*M_dry_*) and volume (*V_dry_* = length ∗ width ∗ thickness) were measured, and then they were soaked in distilled water at room temperature for at least 24 h. Thereafter, the samples were extracted, rapidly dried with a paper tissue to remove surface droplets, and their weight (*_Mwet_*_)_ and volume (*V_wet_*) were rapidly measured. Consequently, the water uptake (*wu*, wt %) of each sample was calculated as follows:

$$wu\;\left(wt\;\%\right)=\frac{M_{wet}-M_{dry}}{\;M_{dry}}*100$$

while volume swelling was determined using the following equation:

$$\Delta V\;\left(\%\right)=\frac{V_{wet}-V_{dry}}{\;V_{dry}}*100$$

Swelling tests were also conducted under increasing temperatures [53]. In this case, a separate piece of membrane was immersed in distilled water equilibrated at the appropriate temperatures of 30, 40, 50, 60, 70, or 80 °C. After 2 h, the samples were removed, the surface droplets quickly dried, and the wet mass and volume were measured.

The ^1^H NMR measurements were performed using a Bruker AVANCE 300 wide-bore spectrometer (Bruker Corporation, Billerica, MA, USA) operating at 300 MHz and using ^1^H nuclei. The spectrometer was assembled with a Diff30 Z-diffusion 30 G/cm/A multinuclear probe with substitutable RF inserts. ^1^H NMR spectra were obtained via Fourier transform elaboration of the resulting free-induction decay (FID) of single π/2 pulse sequences. Pulse length (π/2) for the rf pulse was 10 μs, number of scans was 32, and delay time was 4 s. ^1^H NMR spectra were referenced with the signal of deionized water at δ = 0.00 ppm. Specifically, the ^1^H NMR spectrum of pure water was acquired (it resonated at 4.79 ppm with respect to TMS), and then its chemical shift was set to 0 ppm (please see Figure S1) [14,54]. Thereafter, a different NMR tube containing an sPSU or sPL sample was placed in the probe and analyzed. This allowed for a more rapid evaluation of how the nanoconfinement effect and interaction with the functional groups of both the polymer and the filler particles affect the chemical shift of water molecules [55,56]. Signal deconvolution was carried out using Origin Software, and details on mathematical model are provided in the SI. The self-diffusion coefficients (D) of hydroxide ions were measured using Pulsed Field Gradient Stimulated-Echo (PFG-STE) sequence [57], which consists of three 90 °RF pulses (π/2-τ_1_-π/2-τ_m_-π/2) and two gradient pulses applied after the first and third RF pulses. An echo was found at time τ = 2 τ_1_ + τ_m_. Fourier transform (FT) echo decays were examined by means of the relevant Stesjkal–Tanner equation:

$$I=I_{0}e^{-D{\left(\gamma g\delta \right)}^{2}\left(\Delta -\frac{\delta }{3}\right)}$$

where *I* and *I*_0_ are the signal intensity/area with and without a gradient, respectively; *D* is the diffusion coefficient; *γ* is the gyromagnetic ratio; g is the field gradient; *δ* is gradient pulse duration; and Δ is time delay. The experimental parameters were *δ* = 1 ms and Δ = 10 ms, while the gradient amplitude ranged between 100 and 900 G/cm, and it was incremented in 10 steps. The spin–lattice relaxation times (T_1_) were measured using the inversion recovery sequence (π-τ-π/2). Measurements were carried out in the temperature range of 20–130 °C (with a new measurement taken after every 20 °C temperature change) and by leaving the samples to equilibrate for about 15 min at each temperature. The NMR samples were prepared according to a procedure reported elsewhere [58]. The uncertainty in the NMR measurements was ~3%.

A homemade two-electrode cell was employed to measure the through-plane proton conductivity (σ) of the prepared PEMs [33,59]. Circularly shaped samples were cut from the membranes, sandwiched between two sheets of conductive carbon paper, and placed between two graphite blocking electrodes. The cell was placed between the anode and cathode flow field of a fuel-cell-testing device (850C, Scribner Associates, Inc., Southern Pines, NC, USA). The AC impedance responses of the cells were recorded using a PGSTAT 30 potentiostat/galvanostat (Methrom Autolab, Utrecht, The Netherlands) equipped with an FRA module. Voltage amplitude was 10 mV, and the frequency range was 1 Hz–1 MHz. Nyquist plots were analyzed using Metrohm Autolab NOVA software, and the electrolyte resistance (Rel) was determined according to the high-frequency intersection with the real axis. For the measurements, proton conductivity was measured under various operating temperatures (from 20 °C to 120 °C) and relative humidity values (RH = 20–100%). Activation Energy (Ea) for proton conductivity was calculated from the corresponding Arrhenius plot using the following equation:

$$\ln\sigma =-\frac{\mathrm{Ea}}{R}*\frac{1}{T}$$

Where Ea is the activation energy, R is the gas constant, and T is the temperature (expressed in Kelvin). The equation allows for the acquisition of a straight line plot for ln σ versus $\frac{1}{T}$, for which the slope is $-\frac{\mathrm{Ea}}{R}$.

## 3. Results and Discussion

**Figure 2:**
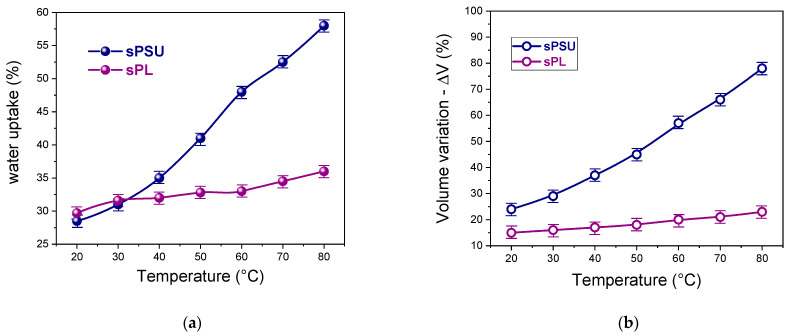
Temperature variation, in the 20–80 °C range, regarding (**a**) water uptake and (**b**) membrane volume for sPSU and sPL membranes.

Figure 2 shows the temperature evolution of water uptake and volume variation for pristine sPSU and sPL nanocomposites. Generally, the hydrophilicity and thus the water uptake of a PEM is mostly governed by its ion exchange capacity (IEC), which provides an indication of the total number of polar groups available for proton transfer [60,61,62]. Anionic lamellae have charged nature; therefore, the introduction of LDH nanoplatelets in the sPES matrix leads to a considerable increase in the IEC of the resulting nanocomposite, i.e., from 1.36 meq g^−1^ for the bare sPSU to 1.49 meq g^−1^. Despite this, at room temperature, there are no significant variations in water uptake with the filler’s incorporation. Indeed, both membranes exhibit a water uptake value of ca. 29% wt. This evidence suggests that the presence of LDH nanoplatelets in the sPSU matrix does not alter the microstructure (i.e., the number, shape, and size) of the hydrophilic clusters but likely affects the distribution of water molecules in such ionic channels.

Further analysis of the swelling behavior under variable temperatures allowed us to gain additional insight into the hydrolytic resistance of the membranes. Indeed, while an adequate amount of water molecules is needed to reach satisfactory proton conductivity, excessive membrane swelling has a detrimental effect on device performance due to rapid MEA deterioration, the mechanical failure of the PEM, and the permeation of non-desirable components through the polymeric membrane [49]. Compared to pristine sPSU, the nanocomposite membrane exhibits superior dimensional resistance. Indeed, both the wu and ΔV for sPSU massively increase with the increase in temperature due to the progressive softening of the polymer chains during heating, which induces an increase in the free volume responsible for membrane over-swelling. Contrarily, the variation in water uptake and membrane dimension are almost negligible for sPL. To better understand this phenomenon, one should consider that LDH lamellae are positively charged. This enables strong electrostatic interactions with negatively charged sulfonic groups of sPSU. The LDH material acts as a physical crosslinker between -SO_3_^−^ groups of adjacent polymer chains, thus preventing any dimensional variation in the ion channels. This provides impressive dimensional stability over a wide range of temperatures. DMA characterization further corroborated this speculation. By observing the variation in the dumping factor (tan δ) vs. temperature illustrated in Figure S2, it can be clearly seen that the Tg of the hydrophilic clusters in sPSU remarkably increases after the introduction of LDH nanoplatelets, i.e., from 200 °C for sPSU to 225 for sPL. This indicates restricted segmental movement due to more intense interaction between sulfonic acid groups of adjacent polymer chains. Such a phenomenon can only be ascribed to physical crosslinking mediated by the LDH nanoplatelets.

To achieve a fundamental understanding of the influence of the LDH nano-additive on the distribution and mobility of water molecules, NMR spectroscopy was extensively used for the investigation of the transport properties of water confined in the hydrophilic clusters of sPSU through direct measurements of ^1^H NMR spectra, self-diffusion coefficients, and spin–lattice relaxation times.

**Figure 3:**
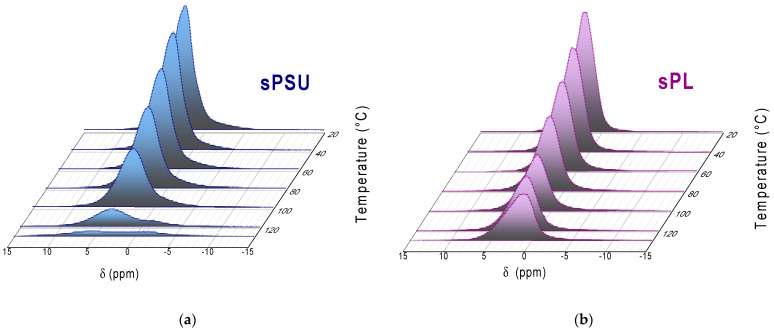

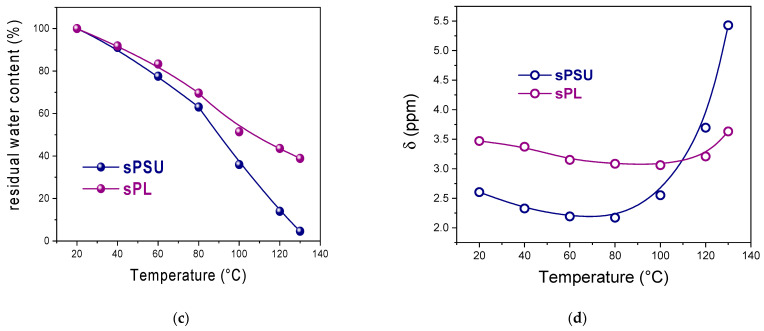
Temperature evolution of high-resolution ^1^H NMR spectra of the water confined in (**a**) sPSU and (**b**) sPL membranes; (**c**) normalized residual water content and (**d**) chemical shift versus temperature of the water 1H spectra acquired for sPSU and sPL membranes.

The temperature evolution within the range of 20–130 °C of the proton spectra acquired for sPSU and sPL is illustrated in Figure 3a,b, respectively. The spectra were referenced against pure water set at 0 ppm and were acquired with the same number of scans to compare their intensities. Clearly, the signals for both sPSU and sPL are very broad, which is a typical indication that water molecules are nanoconfined in both systems. Furthermore, adsorbed water strongly interacts with both the functional groups of the polymer (sulfonic groups) and of the LDH. This leads to a further widening of the ^1^H NMR signals. The variation in the peak area was used to calculate the normalized residual water content vs. temperature for both PEMs, and the results are illustrated in Figure 3c. For temperatures below 80 °C, there is a progressive reduction in water content (to almost 65%) due to gradual evaporation, but both membranes exhibit similar behavior. Contrariwise, they start to diverge in the high-temperature range. While a massive amount of water evaporates from sPSU above 100 °C and the membrane reaches 130 °C in an almost dehydrated state, the water content continuously decreases linearly in the case of sPL. Noteworthily, at 130 °C, the nanocomposite membrane still exhibits a residual water content of 40%, indicating that sPL is able to retain a considerable amount of water even at very high temperatures. The results of the water release tests further confirmed this interesting feature of sPL (see Figure S3). The variation in the chemical shift of the water resonance with temperature might offer significant information on the temperature dependence of liquid water and on the “states” of the water confined inside the membranes. The temperature evolution of the chemical shift for both sPSU and sPL has been illustrated in Figure 3d. It can be observed that the heating causes a downfield shift in resonance, leading to a decrease in the chemical shift. Indeed, thermal energy affects the lifetime of the hydrogen bonds and thus contributes to the proton resonance variation by increasing the mobility of water at a molecular scale as well. Comparatively, sPSU exhibits a minimum at 80 °C, followed by a more marked increase in the chemical shift, which can be clearly related to a non-negligible loss of water due to evaporation. It can be hypothesized that before the minimum is reached, the bulk-like water mostly contributes to the chemical shift, but the residual water remaining at higher temperatures is tightly bound to the acid groups of sPSU. The effect of the fields induced by the hydrophilic polar groups on the temperature is overwhelming, producing an upfield chemical shift. Contrarily, this change in the slope takes place at 100 °C for sPL, presenting a very moderate increase in its chemical shift, indicating that both the bound and bulk-like populations contribute to chemical signal resonance.

**Figure 4:**
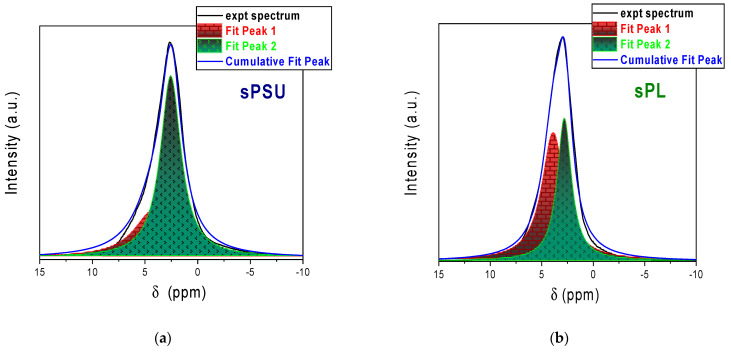
Peak fitting of the ^1^H NMR spectra of the water confined in (**a**) pristine sPSU and (**b**) sPL membranes.

Peak-fitting analysis of the ^1^H NMR spectra was used to definitively clarify the distribution of water molecules inside the investigated systems. As anticipated above, the signal is clearly asymmetric for both membranes, which typically relates to a multicomponent configuration, meaning that water molecules coexist under different “chemical environments”. However, discerning these different “types” of water is not straightforward due to the fast rate of proton exchange in acidic water, and for this reason, we “see” only one peak. Despite this, signal deconvolution can help in clarifying the water distribution and the evaporation dynamics. Indeed, it has been largely demonstrated this technique can be used to successfully elucidate molecular dynamics and confinement effects in complex systems [63,64,65,66,67,68]. In this regard, Figure 4 illustrates the deconvolution of the signals originating from the water molecules adsorbed in the sPSU and sPL nanocomposites. In this case, it can be clearly seen that the signals are properly fitted only by two Lorentzian peaks (one broader (peak-1) and one narrower (peak-2)). Both the chemical shift and the signal linewidth suggest that peak-1 arises from “bound” water, namely, water molecules solvating sulfonic acid groups of sPSU and charged LDH lamellae, while peak-2 can be attributed to highly mobile “bulk” water.

**Figure 5:**
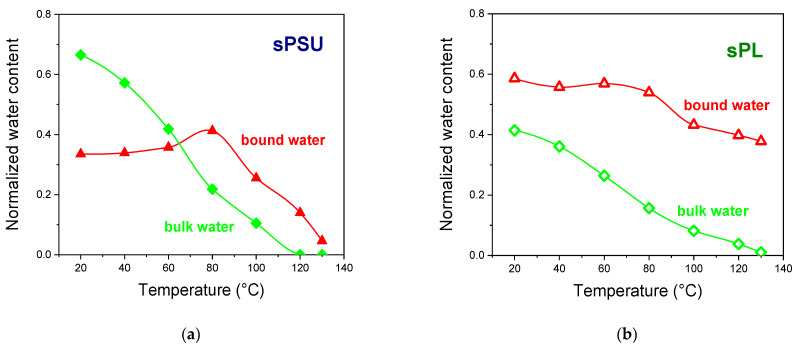
Temperature variation of the two water populations (results from the peak-fitting analysis that have been normalized to the water uptake values) for (**a**) sPSU and (**b**) sPL.

By considering the area of these two peaks, the amounts of bulk and bound water were calculated at each temperature (normalized to the relative water uptake). In this regard, Figure 5 shows the temperature variation of the two “water populations” for both sPSU and sPL membranes. The data point out some crucial differences:

(i) At room temperature, the amount of bulk water in the completely swollen sPSU is surely predominant, whereas the introduction of LDH nanoplatelets induces an impressive increase in the amount of bulk water. This proves that the filler particles only affect the water distribution rather than altering the microstructure of the hydrophilic clusters.
(ii) Water evaporation mostly involves the bulk water, which is more mobile and thus easily evaporates during heating. However, considering the water loss at 100 °C, sPSU loses 64% wt. of the total amount initially absorbed (−55% wt. arising from peak-2; −8% wt. from peak-1). Contrariwise, the water loss for sPL only amounts to 40% wt. From this amount, 25% wt. arises from the bulk population, and 15.2% wt. arises from the solvation shells.
(iii) While the bare polymer reaches 130 °C in an almost dehydrated state, the sPL nanocomposite still contains a considerable amount of water, i.e., ca. 40% wt., which exclusively arises from the water population in the bound state. This suggests water molecules experience strong electrostatic interactions with the LDH platelets, thereby preventing a considerable degree of water evaporation.

The feature above enables sPL to successfully operate in high-temperature and low-humidity PEMFCs [49].

**Figure 6:**
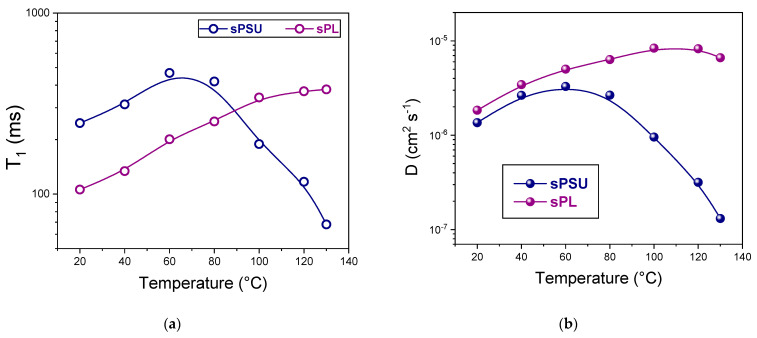
(**a**) Spin–lattice relaxation times (T_1_) and (**b**) self-diffusion coefficients of water confined in sPSU-based membranes as a function of temperature (from 20 °C to 130 °C).

To shed light on the molecular interactions of the water molecules inside the PEM, an extensive analysis of spin–lattice relaxation times (T1) was carried out. T_1_ relates to localized motions, including both translation and rotation, on a time scale comparable to the reciprocal of the angular frequency of NMR (a few nanoseconds). Consequently, the larger the interactions between spin and lattice, the quicker the relaxation and thus the shorter the T_1_ [69]. The temperature behavior of T_1_ for the sPSU and sPL membranes is illustrated in Figure 6a with regard to the temperature range of 20–130 °C. It should be clarified that the T_1_ value measured here is an average value between the characteristic relaxation times of the two aforementioned water populations. Pristine sPSU exhibits higher T_1_ values in the low temperature range, which then rapidly and remarkably fall above 80 °C. As mentioned above, the water molecules in sPSU mostly exist in a bulk state characterized by lower interactions with the lattice. This water population has greater mobility but is also more susceptible to abrupt evaporation. Consequently, above 100 °C, only water-solvating -SO_3_H groups of the polymer remain in the membrane, which is characterized by restricted rotational and/or translational motions. For this reason, T_1_ decreases. On the contrary, T_1_ constantly increases for the entire temperature range in the case of sPL. This evidence indicates that the water molecules are distributed between the various hydrophilic acid sites of the polymer and the filler, with strong electrostatic interactions that decelerate evaporation even at a high T.

Furthermore, direct measurements of the water self-diffusion coefficients (D) allowed for the elucidation of the effect of the LDH nanoplatelets on the long-range mobility [70]. Figure 6b shows the water self-diffusion coefficients of the membranes measured at their maximum water uptake level in the temperature range of 20–130 °C. From these results, it is clear that the introduction of LDH material determines the transport properties of the resulting nanocomposite membrane: the diffusivity of the sPL membrane exceeded that of sPSU for all the temperature ranges investigated but particularly in the high temperature region. For instance, at 130 °C, the water self-diffusion coefficient of sPL is 6.63 × 10^−6^ cm^2^ s^−1^, which is more than one order of magnitude higher than pristine sPSU (namely, 1.31 × 10^−7^ cm^2^ s^−1^). Such an outstanding feature can be ascribed to a combination of two synergistic factors: (i) LDH nanoplatelets are directly involved in the proton transport mechanism; (ii) as mentioned above, the filler particles enable the retention of a considerable amount of “bound but still mobile” water that ensures high diffusivity events under dehydrating operating conditions, i.e., very high temperatures without any additional humidification. Contrariwise, rapid water evaporation above 80 °C results in a sudden drop in the D values.

**Figure 7:**
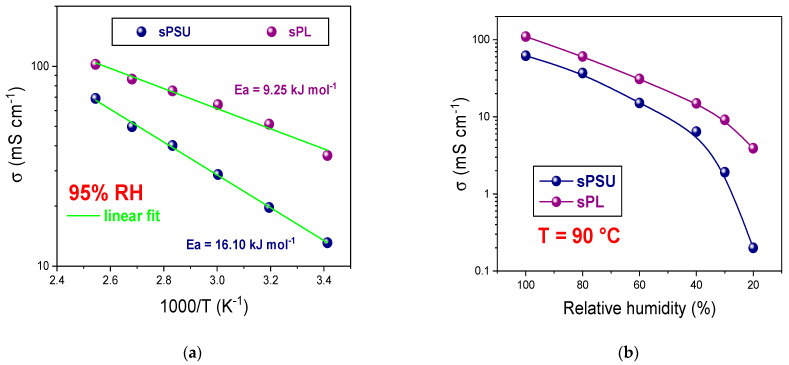
(**a**) Arrhenius plot concerning the proton conductivity of sPSU-based membranes at 95% RH; (**b**) proton conductivity (mS cm^−1^) of sPSU-based membranes at 90 °C as a function of RH.

Finally, the transport properties of the two PEMs were further investigated via electrochemical impedance spectroscopy through measurements of their proton conductivity (σ). Figure 7a illustrates an Arrhenius plot of σ measured from the sPSU and sPL nanocomposites at 95% RH in the temperature range between 20 °C and 120 °C. Bare sPSU yielded a proton conductivity ranging between 13 mS cm^−1^ at 20 °C and 69 mS cm^−1^ at 80 °C, and these findings are in agreement with the literature data [50,71]. As expected, the proton conductivity remarkably increases after the introduction of LDH lamellae, reaching a quite noticeable value of 102 mS cm^−1^ at 120 °C. Such a large enhancement can be ascribed to the fact that 2D nanoplatelets directly contribute to proton transport, likely boosting both the vehicular and Grotthuss mechanisms. The conduction barriers, estimated in the form of activation energies (Ea) calculated from the corresponding Arrhenius plots (see Figure S4), further confirm this assumption. The activation energy decreases from 16.10 kJ mol^−1^ in the case of the bare polymer to 9.25 kJ mol^−1^ due to the incorporation of LDH nanoplatelets, clearly indicating a noticeable improvement in the efficiency of proton conduction. However, the outstanding enhancement in proton conductivity upon the addition of the LDH material is even more evident at lower-humidity conditions. In this regard, Figure 7b shows the humidity dependence of proton conductivity for the sPSU and sPL membranes at 90 °C. As expected, conductivity decreases with decreasing humidity due to a lack of continuous paths for proton conduction along with the progressive depletion of water molecules. However, while sPSU exhibits an abrupt collapse in proton conductivity below 40 % RH, the performance reduction is only moderate in the case of sPL. Notably, the nanocomposite membrane yields a conductivity of ca. 4 mS cm^−1^ at 20 % RH that is 20-fold higher than that of pristine sPSU (i.e., 0.2 mS cm^−1^). This feature is not only due to the impressive water retention capacity of sPL but also to the ability of the LDH material to act as a physical crosslinker. Indeed, the nanoplatelets likely connect isolated sulfonic acid groups, avoiding dead-end pathways and thus generating continuous networks for proton migration. This ensures highly efficient proton conduction through the Grotthuss mechanism, which is dominant under dehydrating conditions. This is a noteworthy result if compared with the conductivity yielded by the Nafion benchmark. Under the same experimental conditions, the conductivity of the Nafion membranes ranges between 155 at 100% mS cm^−1^ RH and 2.8 mS cm^−1^ at 20 % RH [58], which means that sPL is able to exceed the performance of the current benchmark under very harsh operating conditions. This feature is highly desirable for PEMFCs operating at high temperatures and/or low humidity.

## 4. Conclusions

Nanocomposite membranes based on sulfonated polysulfone (sPSU) and Layered Double Hydroxides (LDHs) were prepared via simple solution intercalation and their water molecular dynamics and transport properties were investigated using ^1^H NMR techniques. Swelling tests demonstrated that the presence of the LDH material remarkably enhances the dimensional stability of sPSU by limiting the swelling of its hydrophilic clusters, particularly at high temperatures. Analysis of the ^1^H NMR spectra revealed that two water populations coexist in both PEMs; these populations are water-molecule-solvating functional groups of both the polymer and LDH material (bound water) and molecules in a bulk state. However, the presence of LDH nanoparticles alters their overall distribution. Comparatively, most of the water molecules in sPL are in a bound state. Both the spectral deconvolution data and the T_1_ relaxation times revealed that water molecules experience stronger interactions in sPL that limit evaporation, even at high temperatures, and enable the membrane to retain almost 40% wt of its original water content at 130 °C without any further humidification. Additionally, LDH platelets are directly involved in the proton transport mechanism. The two features enable the sPL membrane to reach a water self-diffusion coefficient of 6.63 × 10^−6^ cm^2^ s^−1^ at 130 °C and, even more importantly, a proton conductivity of almost 4 mS cm^−1^ at 90 °C and 20% RH, constituting a considerable improvement compared to pristine sPSU.

## Data Availability

Not applicable.

## Supplementary Materials

The following supporting information can be downloaded at: https://www.mdpi.com/article/10.3390/membranes13070684/s1. Details on mathematical model used for spectral deconvolution. Figure S1. ^1^H NMR spectra of deionized water. Figure S2. Dynamic mechanical analysis characterization of sPSU and sPL membranes. Figure S3. Water release behaviors of the sPSU-based membranes as a function of temperature. Figure S4. Arrhenius plot (ln σ vs. 1000/T) regarding proton conductivity of sPSU and sPL membranes.

## References

[1] Dijoux E., Steiner N.Y., Benne M., Péra M.C., Pérez B.G. (2017). A Review of Fault Tolerant Control Strategies Applied to Proton Exchange Membrane Fuel Cell Systems. J. Power Sources.

[2] Kim D.J., Jo M.J., Nam S.Y. (2015). A Review of Polymer-Nanocomposite Electrolyte Membranes for Fuel Cell Application. J. Ind. Eng. Chem..

[3] Raja Rafidah R.S., Rashmi W., Khalid M., Wong W.Y., Priyanka J. (2020). Recent Progress in the Development of Aromatic Polymer-Based Proton Exchange Membranes for Fuel Cell Applications. Polymers.

[4] Tellez-Cruz M.M., Escorihuela J., Solorza-Feria O., Compañ V. (2021). Proton Exchange Membrane Fuel Cells (Pemfcs): Advances and Challenges. Polymers.

[5] Sharaf O.Z., Orhan M.F. (2014). An Overview of Fuel Cell Technology: Fundamentals and Applications. Renew. Sustain. Energy Rev..

[6] Peighambardoust S.J., Rowshanzamir S., Amjadi M. (2010). Review of the Proton Exchange Membranes for Fuel Cell Applications.

[7] Cleghorn S.J.C., Ren X., Springer T.E., Wilson M.S., Zawodzinski C., Zawodzinski T.A., Gottesfeld S. (1997). PEM Fuel Cells for Transportation and Stationary Power Generation Applications. Int. J. Hydrogen Energy.

[8] Yang C., Costamagna P., Srinivasan S., Benziger J., Bocarsly A.B. (2001). Approaches and Technical Challenges to High Temperature Operation of Proton Exchange Membrane Fuel Cells. J. Power Sources.

[9] Alberti G., Casciola M., Massinelli L., Bauer B. (2001). Polymeric Proton Conducting Membranes for Medium Temperature Fuel Cells (110–160°C). J. Memb. Sci..

[10] Kim Y.M., Choi S.H., Lee H.C., Hong M.Z., Kim K., Lee H.I. (2004). Organic-Inorganic Composite Membranes as Addition of SiO_2_ for High Temperature-Operation in Polymer Electrolyte Membrane Fuel Cells (PEMFCs). Electrochim. Acta.

[11] Okonkwo P.C., Ben Belgacem I., Emori W., Uzoma P.C. (2021). Nafion Degradation Mechanisms in Proton Exchange Membrane Fuel Cell (PEMFC) System: A Review. Int. J. Hydrogen Energy.

[12] Sopian K., Wan Daud W.R. (2006). Challenges and Future Developments in Proton Exchange Membrane Fuel Cells. Renew. Energy.

[13] Zare A., Montané X., Reina J.A., Giamberini M., Tylkowski B., Wieszczycka K., Jastrząb R., Montane X. (2023). Applications of Membranes in Sustainable Energy Systems: Energy Production and Storage. Polymer Engineering.

[14] Antonucci P.L., Aricò A.S., Cretì P., Ramunni E., Antonucci V. (1999). Investigation of a Direct Methanol Fuel Cell Based on a Composite Nafion-Silica Electrolyte for High Temperature Operation. Solid State Ionics.

[15] Adjemian K.T., Dominey R., Krishnan L., Ota H., Majsztrik P., Zhang T., Mann J., Kirby B., Gatto L., Velo-Simpson M. (2006). Function and Characterization of Metal Oxide−Nafion Composite Membranes for Elevated-Temperature H_2_/O_2_ PEM Fuel Cells. Chem. Mater..

[16] Mishra A.K., Bose S., Kuila T., Kim N.H., Lee J.H. (2012). Silicate-Based Polymer-Nanocomposite Membranes for Polymer Electrolyte Membrane Fuel Cells. Prog. Polym. Sci..

[17] Del Río C., Morales E., Escribano P.G. (2014). Nafion/SPOSS Hybrid Membranes for PEMFC. Single Cell Performance and Electrochemical Characterization at Different Humidity Conditions. Int. J. Hydrogen Energy.

[18] D’Epifanio A., Navarra M.A., Weise F.C., Mecheri B., Farrington J., Licoccia S., Greenbaum S. (2010). Composite Nafion/Sulfated Zirconia Membranes: Effect of the Filler Surface Properties on Proton Transport Characteristics. Chem. Mater..

[19] Nicotera I., Simari C., Boutsika L.G., Coppola L., Spyrou K., Enotiadis A. (2017). NMR Investigation on Nanocomposite Membranes Based on Organosilica Layered Materials Bearing Different Functional Groups for PEMFCs. Int. J. Hydrogen Energy.

[20] Enotiadis A., Boutsika L.G., Spyrou K., Simari C., Nicotera I. (2017). A Facile Approach to Fabricating Organosilica Layered Material with Sulfonic Groups as an Efficient Filler for Polymer Electrolyte Nanocomposites. New J. Chem..

[21] Branchi M., Sgambetterra M., Pettiti I., Panero S., Navarra M.A. (2015). Functionalized Al_2_O_3_ Particles as Additives in Proton-Conducting Polymer Electrolyte Membranes for Fuel Cell Applications. Int. J. Hydrogen Energy.

[22] Chien H.C., Tsai L.D., Huang C.P., Kang C.Y., Lin J.N., Chang F.C. (2013). Sulfonated Graphene Oxide/Nafion Composite Membranes for High-Performance Direct Methanol Fuel Cells. Int. J. Hydrogen Energy.

[23] Kumar R., Xu C., Scott K. (2012). Graphite Oxide/Nafion Composite Membranes for Polymer Electrolyte Fuel Cells. RSC Adv..

[24] Shao Z.G., Xu H., Li M., Hsing I.M. (2006). Hybrid Nafion-Inorganic Oxides Membrane Doped with Heteropolyacids for High Temperature Operation of Proton Exchange Membrane Fuel Cell. Solid State Ionics.

[25] Zhang Y., Fei X., Zhang G., Li H., Shao K., Zhu J., Zhao C., Liu Z., Han M., Na H. (2010). Preparation and Properties of Epoxy-Based Cross-Linked Sulfonated Poly(Arylene Ether Ketone) Proton Exchange Membrane for Direct Methanol Fuel Cell Applications. Int. J. Hydrogen Energy.

[26] Zhao C., Lin H., Na H. (2010). Novel Cross-Linked Sulfonated Poly (Arylene Ether Ketone) Membranes for Direct Methanol Fuel Cell. Int. J. Hydrogen Energy.

[27] Kim N.H., Mishra A.K., Kim D.Y., Lee J.H. (2015). Synthesis of Sulfonated Poly(Ether Ether Ketone)/Layered Double Hydroxide Nanocomposite Membranes for Fuel Cell Applications. Chem. Eng. J..

[28] Sonpingkam S., Pattavarakorn D. (2014). Mechanical Properties of Sulfonated Poly (Ether Ether Ketone) Nanocomposite Membranes. Int. J. Chem. Eng. Appl..

[29] Kawaguti C.A., Dahmouche K., Gomes A.d.S. (2012). Nanostructure and Properties of Proton-Conducting Sulfonated Poly(Ether Ether Ketone) (SPEEK) and Zirconia-SPEEK Hybrid Membranes for Direct Alcohol Fuel Cells: Effect of the Nature of Swelling Solvent and Incorporation of Heteropolyacid. Polym. Int..

[30] You P.Y., Kamarudin S.K., Masdar M.S. (2019). Improved Performance of Sulfonated Polyimide Composite Membranes with Rice Husk Ash as a Bio-Filler for Application in Direct Methanol Fuel Cells. Int. J. Hydrogen Energy.

[31] Miyatake K., Furuya H., Tanaka M., Watanabe M. (2012). Durability of Sulfonated Polyimide Membrane in Humidity Cycling for Fuel Cell Applications. J. Power Sources.

[32] Li J., Wu H., Cao L., He X., Shi B., Li Y., Xu M., Jiang Z. (2019). Enhanced Proton Conductivity of Sulfonated Polysulfone Membranes under Low Humidity via the Incorporation of Multifunctional Graphene Oxide. ACS Appl. Nano Mater..

[33] Simari C., Lufrano E., Brunetti A., Barbieri G., Nicotera I. (2021). Polysulfone and Organo-Modified Graphene Oxide for New Hybrid Proton Exchange Membranes: A Green Alternative for High-Efficiency PEMFCs. Electrochim. Acta.

[34] Maier G., Meier-Haack J. (2008). Sulfonated Aromatic Polymers for Fuel Cell Membranes. Adv. Polym. Sci..

[35] Ozden A., Ercelik M., Devrim Y., Colpan C.O., Hamdullahpur F. (2017). Evaluation of Sulfonated Polysulfone/Zirconium Hydrogen Phosphate Composite Membranes for Direct Methanol Fuel Cells. Electrochim. Acta.

[36] Lufrano F., Squadrito G., Patti A., Passalacqua E. (2000). Sulfonated Polysulfone as Promising Membranes for Polymer Electrolyte Fuel Cells. J. Appl. Polym. Sci..

[37] Devrim Y., Erkan S., Baç N., Eroǧlu I. (2009). Preparation and Characterization of Sulfonated Polysulfone/Titanium Dioxide Composite Membranes for Proton Exchange Membrane Fuel Cells. Int. J. Hydrogen Energy.

[38] Simari C., Prejanò M., Lufrano E., Sicilia E., Nicotera I. (2021). Exploring the Structure–Performance Relationship of Sulfonated Polysulfone Proton Exchange Membrane by a Combined Computational and Experimental Approach. Polymers.

[39] Rives V., Rives V. (2001). Layered Double Hydroxides: Present and Future.

[40] Miyata S. (1983). Anion-Exchange Properties of Hydrotalcite-like Compounds. Clays Clay Miner..

[41] Cavani F., Trifirò F., Vaccari A. (1991). Hydrotalcite-Type Anionic Clays: Preparation, Properties and Applications. Catal. Today.

[42] Aramendía M.A., Borau V., Jiménez C., Marinas J.M., Ruiz J.R., Urbano F.J. (2001). Catalytic Transfer Hydrogenation of Citral on Calcined Layered Double Hydroxides. Appl. Catal. A Gen..

[43] Vaccari A. (1998). Preparation and Catalytic Properties of Cationic and Anionic Clays. Catal. Today.

[44] Sels B.F., De Vos D.E., Jacobs P.A. (2001). Hydrotalcite-like Anionic Clays in Catalytic Organic Reactions. Catal. Rev.-Sci. Eng..

[45] Climent M.J., Corma A., Iborra S., Primo J. (1995). Base Catalysis for Fine Chemical Production: Claisen-Schmidt Condensation on Zeolites and Hydrocalcites for the Production of Chalcones and Flavanones of Pharmaceutical Interes. J. Catal..

[46] Oestreicher V., Jobbágy M., Regazzoni A.E. (2014). Halide Exchange on Mg(II)-Al(III) Layered Double Hydroxides: Exploring Affinities and Electrostatic Predictive Models. Langmuir.

[47] Herrero M., Martos A.M., Varez A., Galván J.C., Levenfeld B. (2014). Synthesis and Characterization of Polysulfone/Layered Double Hydroxides Nanocomposite Membranes for Fuel Cell Application. Int. J. Hydrogen Energy.

[48] Simari C., Lufrano E., Brunetti A., Barbieri G., Nicotera I. (2020). Highly-Performing and Low-Cost Nanostructured Membranes Based on Polysulfone and Layered Doubled Hydroxide for High-Temperature Proton Exchange Membrane Fuel Cells. J. Power Sources.

[49] Lufrano E., Simari C., Lo Vecchio C., Aricò A.S., Baglio V., Nicotera I. (2020). Barrier Properties of Sulfonated Polysulfone/Layered Double Hydroxides Nanocomposite Membrane for Direct Methanol Fuel Cell Operating at High Methanol Concentrations. Int. J. Hydrogen Energy.

[50] Lufrano F., Gatto I., Staiti P., Antonucci V., Passalacqua E. (2001). Sulfonated Polysulfone Ionomer Membranes for Fuel Cells. Solid State Ionics.

[51] Gayathri R., Prabhu M.R. (2020). Protonated State and Synergistic Role of Nd^3+^ doped Barium Cerate Perovskite for the Enhancement of Ionic Pathways in Novel Sulfonated Polyethersulfone for H_2_/O_2_ fuel Cells. Soft Matter.

[52] Simari C., Lo Vecchio C., Baglio V., Nicotera I. (2020). Sulfonated Polyethersulfone/Polyetheretherketone Blend as High Performing and Cost-Effective Electrolyte Membrane for Direct Methanol Fuel Cells. Renew. Energy.

[53] Nicotera I., Policicchio A., Conte G., Giuseppe R., Habib M., Rehman U., Lufrano E., Simari C. (2022). Quaternized Polyepichlorohydrin-Based Membrane as High-Selective CO_2_ Sorbent for Cost-Effective Carbon Capture. J. CO_2_ Util..

[54] Lufrano E., Simari C., Enotiadis A., Nicotera I. (2022). Sulfonated Polyether Ether Ketone and Organosilica Layered Nanofiller for Sustainable Proton Exchange Membranes Fuel Cells (PEMFCs). Appl. Sci..

[55] Lufrano E., Nicotera I., Enotiadis A., Rehman M.H.U., Simari C. (2022). Elucidating the Water and Methanol Dynamics in Sulfonated Polyether Ether Ketone Nanocomposite Membranes Bearing Layered Double Hydroxides. Membranes.

[56] Simari C., Enotiadis A., Nicotera I. (2020). Transport Properties and Mechanical Features of Sulfonated Polyether Ether Ketone/Organosilica Layered Materials Nanocomposite Membranes for Fuel Cell Applications. Membranes.

[57] Stejskal E.O., Tanner J.E. (1965). Spin Diffusion Measurements: Spin Echoes in the Presence of a Time-Dependent Field Gradient. J. Chem. Phys..

[58] Simari C., Lufrano E., Godbert N., Gournis D., Coppola L., Nicotera I. (2020). Titanium Dioxide Grafted on Graphene Oxide: Hybrid Nanofiller for Effective and Low-Cost Proton Exchange Membranes. Nanomaterials.

[59] Miyatake K., Zhou H., Matsuo T., Uchida H., Watanabe M. (2004). Proton Conductive Polyimide Electrolytes Containing Trifluoromethyl Groups: Synthesis, Properties, and DMFC Performance. Macromolecules.

[60] Yee R.S.L., Zhang K., Ladewig B.P. (2013). The Effects of Sulfonated Poly(Ether Ether Ketone) Ion Exchange Preparation Conditions on Membrane Properties. Membranes.

[61] Heo Y., Im H., Kim J. (2013). The Effect of Sulfonated Graphene Oxide on Sulfonated Poly (Ether Ether Ketone) Membrane for Direct Methanol Fuel Cells. J. Memb. Sci..

[62] Qiu M., Zhang B., Wu H., Cao L., He X., Li Y., Li J., Xu M., Jiang Z. (2019). Preparation of Anion Exchange Membrane with Enhanced Conductivity and Alkaline Stability by Incorporating Ionic Liquid Modi Fi Ed Carbon Nanotubes. J. Memb. Sci..

[63] Nicotera I., Simari C., Coppola L., Zygouri P., Gournis D., Brutti S., Minuto F.D., Aricò A.S., Sebastian D., Baglio V. (2014). Sulfonated Graphene Oxide Platelets in Nafion Nanocomposite Membrane: Advantages for Application in Direct Methanol Fuel Cells. J. Phys. Chem. C.

[64] Nicotera I., Kosma V., Simari C., D’Urso C., Aricò A.S., Baglio V. (2015). Methanol and Proton Transport in Layered Double Hydroxide and Smectite Clay-Based Composites: Influence on the Electrochemical Behavior of Direct Methanol Fuel Cells at Intermediate Temperatures. J. Solid State Electrochem..

[65] Nicotera I., Kosma V., Simari C., Ranieri G.A., Sgambetterra M., Panero S., Navarra M.A. (2015). An NMR Study on the Molecular Dynamic and Exchange Effects in Composite Nafion/Sulfated Titania Membranes for PEMFCs. Int. J. Hydrogen Energy.

[66] Simari C., Nicotera I., Perrotta I.D., Clarizia G., Bernardo P. (2020). Microscopic and Macroscopic Investigation on the Gas Diffusion in Poly(Ether-Block-Amide) Membranes Doped with Polysorbate Nonionic Surfactants. Polymer.

[67] Simari C., Tuccillo M., Brutti S., Nicotera I. (2022). Sodiated Nafion Membranes for Sodium Metal Aprotic Batteries. Electrochim. Acta.

[68] Policicchio A., Conte G., Agostino R.G., Caputo P., Oliviero Rossi C., Godbert N., Nicotera I., Simari C. (2022). Hexagonal Mesoporous Silica for Carbon Capture: Unrevealing CO_2_ Microscopic Dynamics by Nuclear Magnetic Resonance. J. CO_2_ Util..

[69] Slichter C. (1990). Principles of Magnetic Resonance.

[70] Cossari P., Pugliese M., Simari C., Mezzi A., Maiorano V., Nicotera I., Gigli G. (2020). Simplified All-Solid-State WO_3_ Based Electrochromic Devices on Single Substrate: Toward Large Area, Low Voltage, High Contrast, and Fast Switching Dynamics. Adv. Mater. Interfaces.

[71] Naim R., Ismail A.F., Saidi H., Saion E. (2004). Development of Sulfonated Polysulfone Membranes as a Material for Proton Exchange Membrane (PEM). Proc. Reg. Symp. Membr. Sci. Technol..

