# Supplementary material for: NMR Investigation of Water Molecular Dynamics in Sulfonated Polysulfone/Layered Double Hydroxide Composite Membranes for Proton Exchange Membrane Fuel Cells
Source: Membranes (Basel). 2023 Jul 22;13(7):684. doi: 10.3390/membranes13070684 (PMC10384311; doi:10.3390/membranes13070684)
Supplement: Supplementary file 1 [file membranes-13-00684-s001.zip › membranes-2482559-supplementary.pdf]

Supplementary Information

NMR Investigation of Water Molecular Dynamics in Sulfonated Polysulfone/Layered Double Hydroxide Composite Membranes for PEMFCs

Cataldo Simari [1,2]

[1] Department of Chemistry and Chemical Technologies, University of Calabria, 87036 Rende, Italy; Tel: +39-0984-493385; Fax: +39-0984-492044

[2] National Reference Centre for Electrochemical Energy Storage (GISEL)—INSTM, Via G. Giusti 9, 50121 Firenze, Italy

Spectral deconvolution has been carried out using Lorentzian peak function with the following characteristics:

Number of Parameters=4

Number of Independent Variables=1 (x)

Number of Dependent Variables=1 (y)

[FORMULA] => y = y0 + (2*A/pi)*(w/(4*(x-xc)^2 + w^2))

[Parameters Initialization] => xc = peak_pos(x_y_curve, &w, &y0, NULL, &A);

A *= 1.57*w;

Number of Duplicates=1

Duplicate Offset=2

Duplicate Unit=3

Peak Center=2

Peak Width=3

Peak Amplitude=4

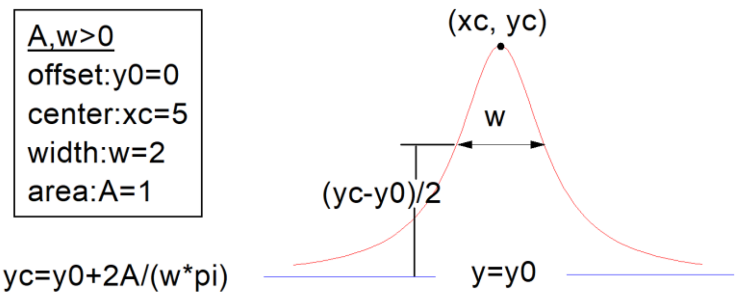

Noteworthy, R2 value for the various spectral deconvolution ranged between 0.9943 and 0.9985 as a clear indication of the goodness of the mathematical fitting model used in this study.

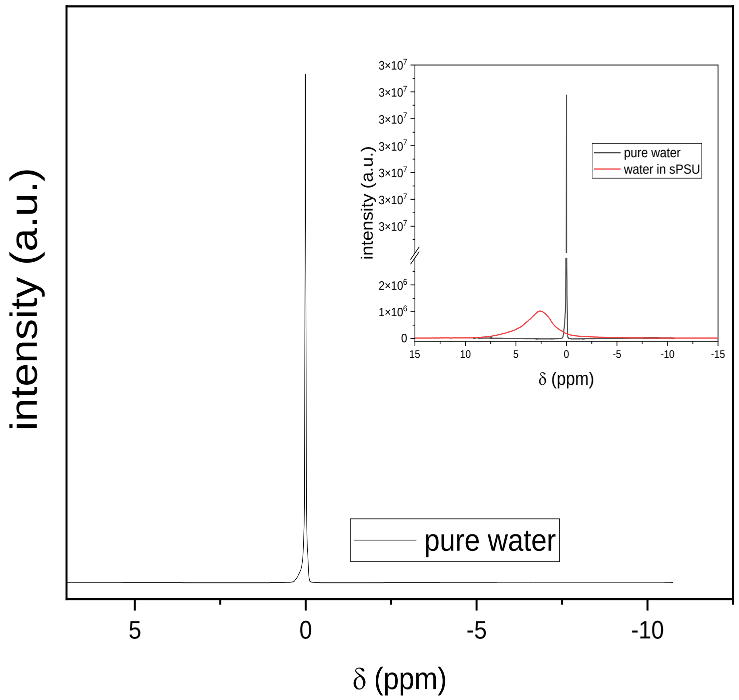

Figure S1. ^{1}H NMR spectra of pure water.

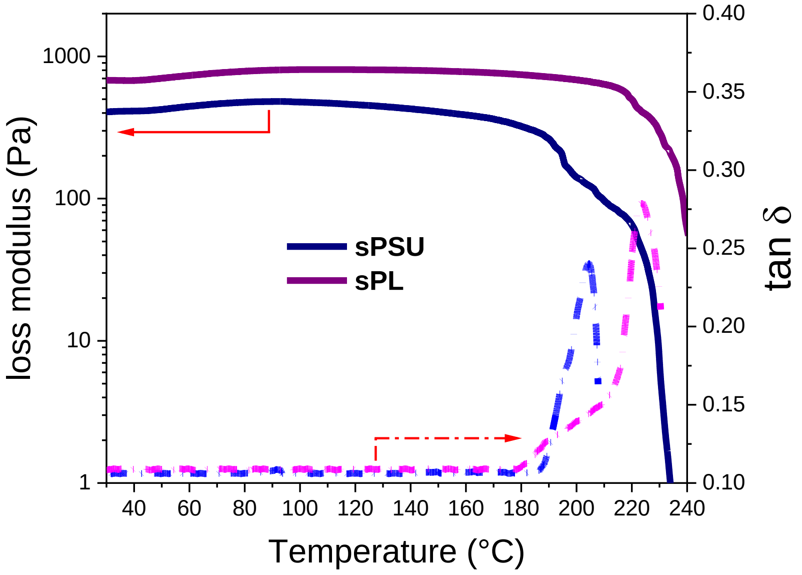

Figure S2. Dynamic Mechanical Analysis characterization on sPSU and sPL membranes

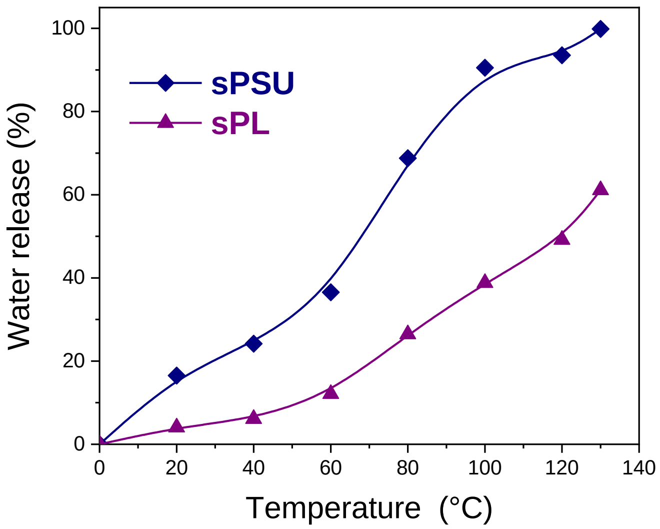

Figure S3. Water release behaviors of the sPSU-based membranes as a function of the temperature.

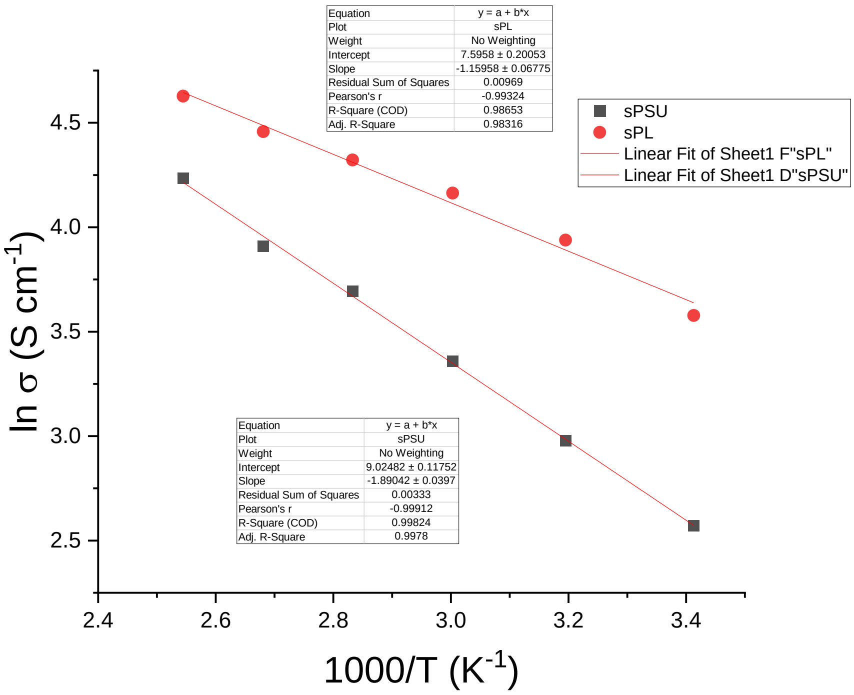

Figure S4. Arrhenius plot (ln σ *vs.* 1000/T) for proton conductivity of sPSU and sPL membranes
